# Case report: A left forearm mass with eccentric intramedullary ulnar destruction diagnosed as alveolar rhabdomyosarcoma and treated by wide resection and free vascularized fibular graft

**DOI:** 10.3389/fonc.2024.1395233

**Published:** 2024-05-10

**Authors:** Chenyu Yang, Xinjia Wang, Huaitai Lin, Jinhao Zhu, Zijian Xu, Weidong Wang

**Affiliations:** Bone and Soft Tissue Tumor Department, Shantou University Medical College Affiliated Cancer Hospital, Shantou, China

**Keywords:** alveolar rhabdomyosarcoma, bone destruction, bone reconstruction, en bloc, MDT

## Abstract

**Background:**

Alveolar Rhabdomyosarcoma is a profoundly malignant soft-tissue sarcoma that predominantly affects children and adolescents. However, the medical field lacks consensus regarding the optimal surgical approach to be undertaken in cases where this tumor causes local bone destruction in the upper limb.

**Case description:**

A 17-year-old male presented a mass in his left forearm and CT and MRI indicated that the mass had penetrated the ulnar cortex and infiltrating the medulla, resulting in the formation of an eccentric trans-ventricular tumor focus. The sizable tumor affected the volar muscles of the forearm as well as the ulnar bone marrow, exerting pressure on the ulnar artery and vein. It was confirmed by needle biopsy that the mass is alveolar rhabdomyosarcoma. Following two courses of neoadjuvant chemotherapy, the tumor was widely excised en bloc. Autologous fibula with a vascular pedicle was utilized for reconstruction during the procedure. In the postoperative follow-up, no local recurrence of the tumor was observed. Furthermore, the patient retained satisfactory wrist flexion and pronation function in the left forearm.

**Conclusions:**

Alveolar rhabdomyosarcoma is an uncommon and highly aggressive form of soft tissue sarcoma. Scientific management necessitates a multidisciplinary approach, combining chemotherapy with surgery. In cases where the tumor invaded into compartment of the bone, careful consideration should be given to the boundaries of tumor resection, the extent of osteotomy, and the approach to musculoskeletal reconstruction when designing the surgical plan. Through reporting our own case and thoroughly reviewing previous clinical experiences, we aim to provide valuable insights for the treatment of this particular disease.

## Introduction

**Figure 1:**
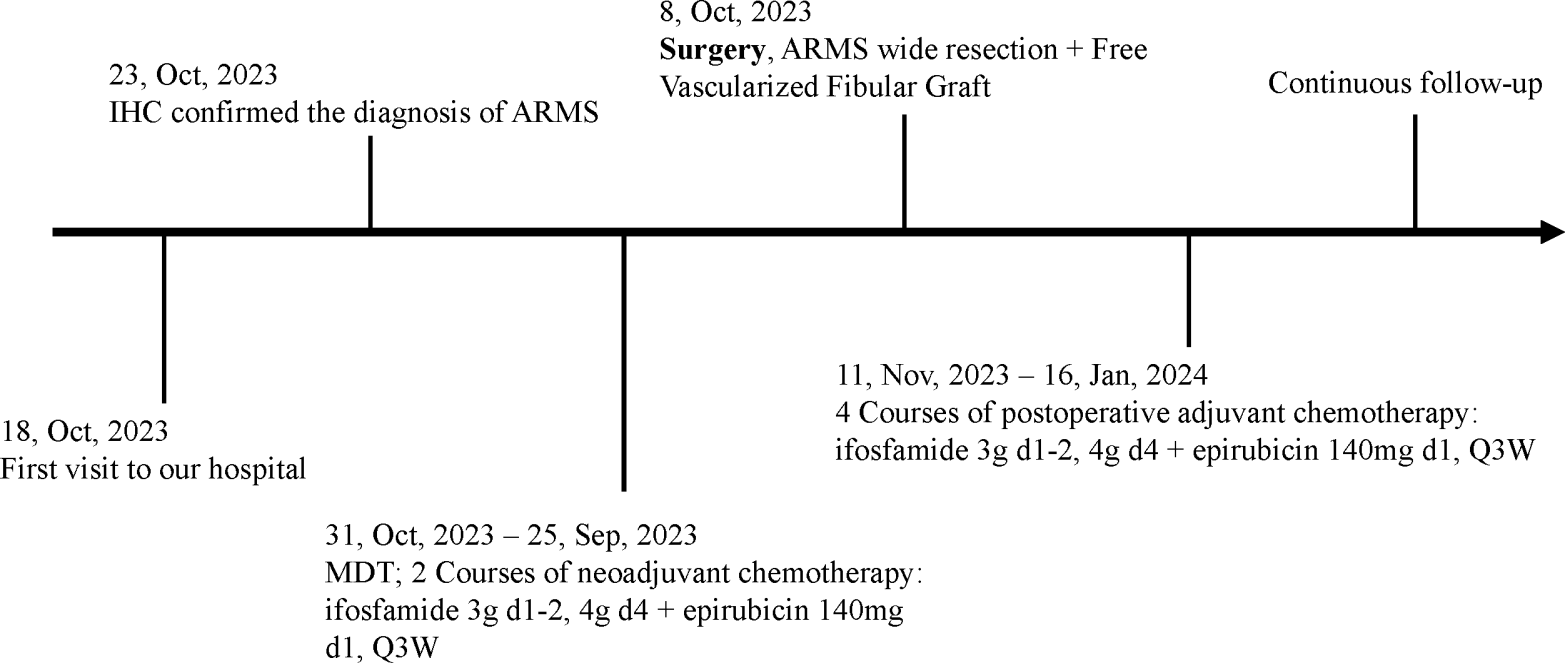
Specific time points corresponding to the diagnostic and therapeutic process.

Rhabdomyosarcoma (RMS), the most prevalent soft tissue sarcoma among children and adolescents, is an extremely malignant sarcoma that exhibits a myogenic differentiation propensity. Annually, there are approximately 6 cases per million individuals worldwide, with the majority affecting children, constituting 4.5% of all childhood cancer cases. The male to female ratio falls between 1.4 to 1.7, and the primary sites include the head and neck, trunk, limbs, and genitourinary system. Moreover, due to the lack of specific clinical symptoms, RMS is frequently diagnosed at advanced stages (III to IV) initially, and early metastasis is possible (1). ARMS, the most undifferentiated subtype of RMS, is associated with aggressive behavior. It is predominantly detected in the extremities, perineum, and paravertebral regions; however, it can potentially occur in any area. Furthermore, this condition may be accompanied by lymph node and bone marrow involvement (2, 3). Several reports have emerged regarding ARMS affecting the upper limbs, particularly in the hands. This condition manifests as a gradually increasing soft tissue mass, typically detected through MRI examination as a uniform solid mass (4, 5). There were also cases of multiple bone marrow involvement after metastasis (6). However, only a small number of cases involving Alveolar Rhabdomyosarcoma (ARMS) have been reported to occur in proximity to the long bone in the forearm. These cases exhibit a distinct pattern of breaking through the compartment and causing localized infiltration and of the bone cortex. In this particular report (specific time points in **Figure 1**), we present a compelling case of ARMS manifestation in the left forearm. Notably, the tumor extensively invaded the volar cortex of the ulna, forcefully penetrating the compartment, and compromising multiple vital flexor muscles situated on the volar surface.

## Case description

### Clinical finding

A 17-year-old Chinese male teenager presented to the orthopedic department with a firm mass in his left forearm that had been causing pain and swelling for the past 2 months. He reported experiencing increased pain in the left forearm after physical activity and weakness in his left hand. He denied any history of injury, fever, or chills. There was no significant past or personal medical history, and laboratory examination did not reveal any obvious abnormalities. During the physical examination, a noteworthy finding was a 5.5×4×2 cm mass located on the ulnar side of the volar surface in the middle part of the left forearm. There were no signs of redness, swelling, ulceration, or varicose veins in the surrounding skin. The patient experienced pain during pronation and supination movements of the left forearm, and there was limited flexion in the left wrist. No abnormalities were detected in the left elbow or the left axillary lymph nodes.

**Figure 2:**
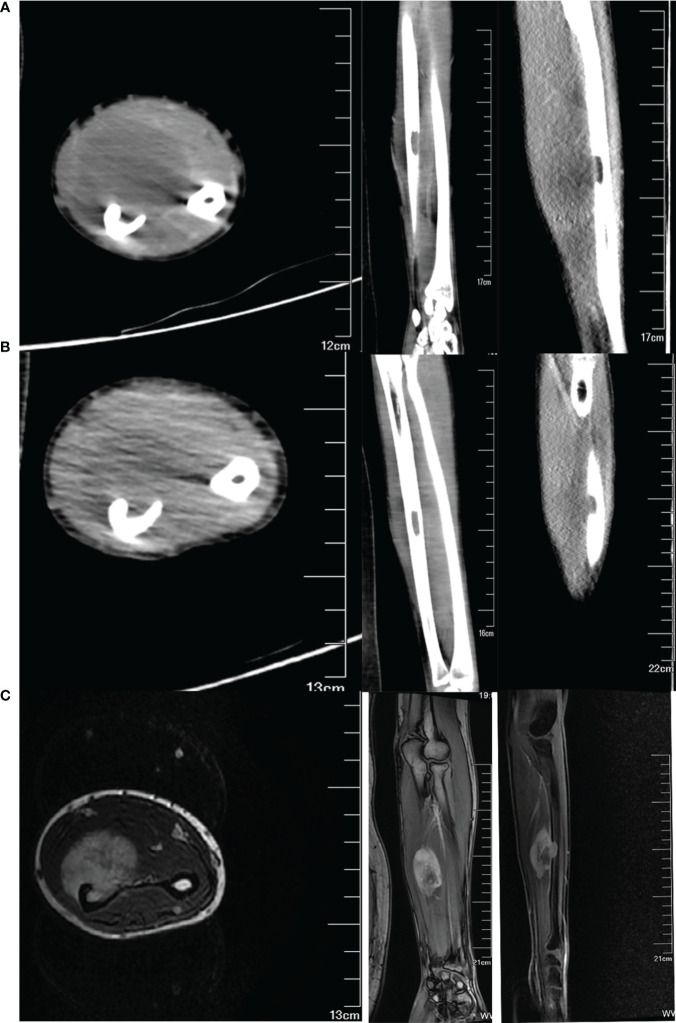
Imaging data of the tumor pre- and post-chemotherapy treatment. **(A)** CT images of the tumor before chemotherapy in transverse, coronal, and sagittal position. **(B)** CT images of the tumor after two courses of chemotherapy. **(C)** T1-weighted MR images of the tumor after two courses of chemotherapy.

The magnetic resonance imaging conducted at another hospital revealed a significant soft tissue mass on the volar side of the left forearm. Upon analysis of T2 weighted images, the mass exhibited uniform hyperintensities. Notably, the interosseous membrane, flexor digitorum profundus, pronator teres, and anterior interosseous artery were all affected to varying extents. Furthermore, the pseudocapsule located on the inner layer of the ulnar artery and radial artery remained intact. The Enhanced CT scan (**Figure 2A**) revealed a 8.7×4.7×3.5 cm soft tissue mass and a 1.7×1 cm focal osteolytic bone destruction on the volar surface of the middle section of the left ulna.

The specimen acquired through percutaneous biopsy indicated the presence of a small round cell tumor. Immunohistochemistry results showed positivity for VIM, MyoD1, Myogenin, Desmin, INT1, Ki67 (80%-90%), Satb2, CD99, and GATA3 (a small amount). Conversely, the staining was negative for CD34, WT1 (except for cytoplasmic staining), EMA, CK, LCA, SMA, S100, HMB45, and SYN. No metastases were identified on the chest and abdomen CT scans or cranial MRI. In conclusion, the patient was ultimately diagnosed with ARMS, classified as cT2N0M0 IIIA.

### Multi-disciplinary treatment

After undergoing a multidisciplinary team (MDT) evaluation, the patient was initially administered neoadjuvant chemotherapy. The initial plan was to utilize the IE regimen, consisting of ifosfamide and etoposide. However, due to the patient’s low tolerance for this regimen, the final decision was to modify it. The revised regimen consisted of ifosfamide 1800 mg/m^2^ on days 1-2, ifosfamide 2400 mg/m^2^ on day 3 and epirubicin 85 mg/m^2^ on day 1. Following a 2-week break, the regimen was repeated once more.

After undergoing two courses of neoadjuvant chemotherapy, the mass in the left forearm notably diminished. Additionally, there was partial relief from pain and an improvement in the range of motion for rotating the left forearm. The MRI (**Figure 2C**) and CT (**Figure 2B**) scan revealed that there was a maximum reduction of approximately 50% in the cross-sectional tumor area, and the extent of involvement in the flexor digitorum profundus and pronator rotundus muscles significantly decreased. Based on our experience in preoperative planning, the tumor must be resected with strict adherence to the en bloc method, ensuring that the resection margin extends to the normal tissue beyond the reaction zone. Furthermore, it is imperative to include a substantial anatomical barrier, such as fascia, within the resection margin. The osteotomy is typically performed within a range of 3-5cm from the intramedullary tumor.

**Figure 3:**
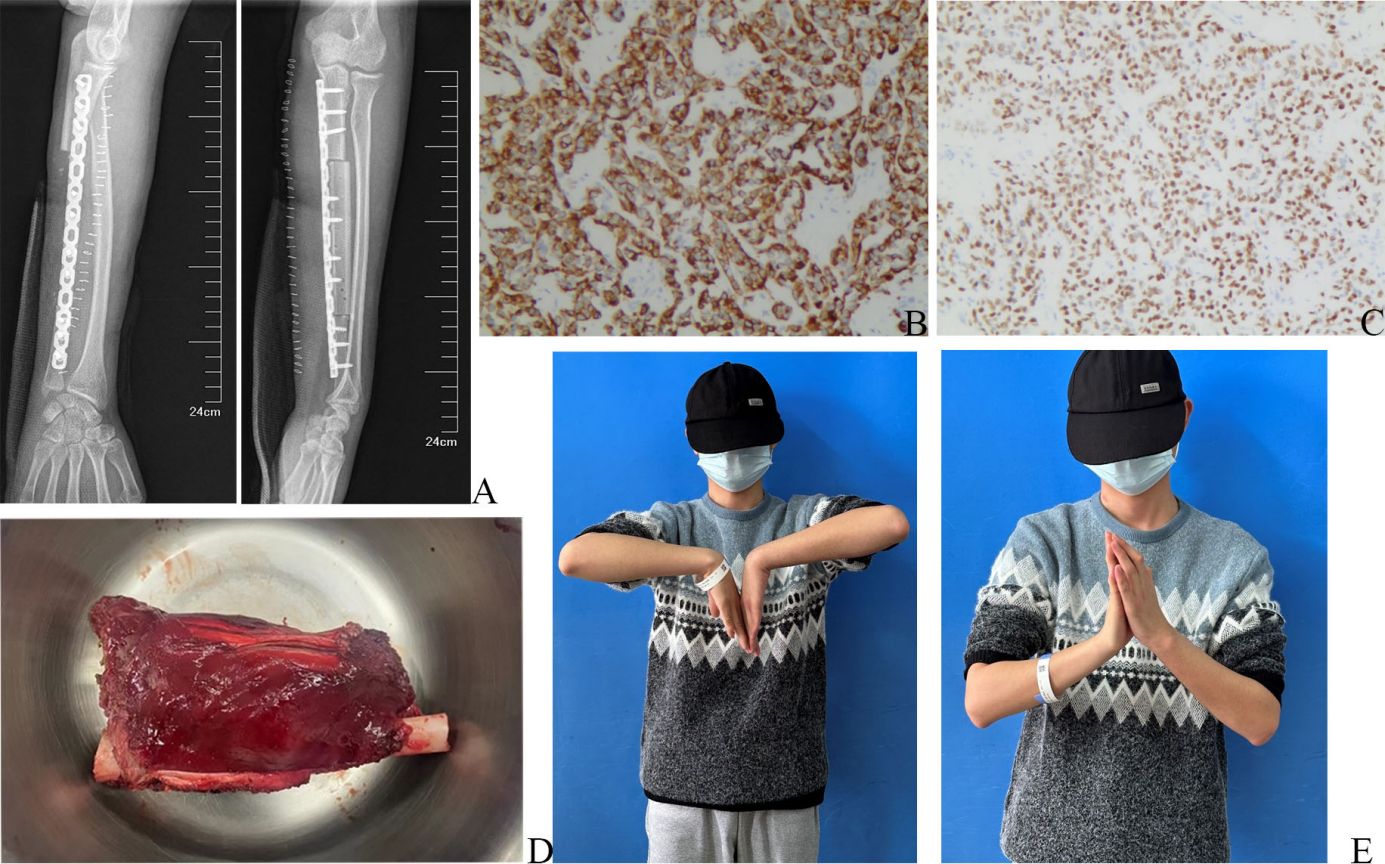
Postoperative X-ray, postoperative pathological pictures, and physical examination of the wrist joint. **(A)** Postoperative X-ray of left forearm. **(B)** The immunohistochemical results of Desmin, under a 100x microscope. **(C)** The immunohistochemical results of MyoD1, under a 100x microscope. **(D)** Intraoperative image of the en bloc tumor. **(E)** Postoperative physical examination.

In surgery, the incision measured approximately 20 cm, and a 1 cm section of the skin surrounding the initial puncture site was carefully secured. Throughout the procedure, utmost care was taken to ensure the safety and freedom of the median nerve, ulnar nerve, palmaris longus, and other vital structures. To remove the soft tissue mass and the ulnar tumor segment, they were excised together in one piece, en bloc (**Figure 3D**), from the flexor digitorum profundus, roughly 3cm away from the pseudocapsule. Notably, the ulnar origin of the extensor pollicis longus and extensor digitorum was appropriately marked for reference. A vascularized fibular graft was procured from the right lower leg and skillfully fixed to the ulna defect using a dependable locking plate. With the aid of a microscope, the blood vessels were meticulously anastomosed, ensuring proper circulation. The damaged muscle was effectively repaired using high-strength sutures at the previously marked point.

### Postoperative pathology and follow-up

The postoperative X-ray examination revealed that the bone graft and internal fixation were in good position (**Figure 3A**). The pathological specimen showed a residual tumor of approximately 70%, consisting mostly of hyalinized and necrotic tissue, which accounted for approximately 30%. Additionally, a vascular tumor thrombus was observed in the bone marrow cavity, while no tumor was detected in the basal, medial, lateral, proximal, and distal sections of the surgical field. Subsequent postoperative immunohistochemistry analysis exhibited positivity for Desmin (**Figure 3B**) and MyoD1 (**Figure 3C**), indicating the presence of these markers in the tumor. However, CD99 and FLI-1 showed negativity, suggesting their absence in the tumor. Focal positivity for SATB2 was also observed, supporting the diagnosis of ARMS.

The patient successfully underwent surgery and proceeded with the preoperative neoadjuvant chemotherapy regimen. Following the procedure, functional exercise was initiated during the second week post-surgery. Notably, the left wrist joint demonstrated a flexion range of motion (ROM) of approximately 90°, while the extension ROM was slightly limited to 60° (**Figure 3E**). Additionally, the wrist flexor muscle strength was rated at grade V, while the finger flexor muscle strength was also grade V. The finger extensor muscle strength was graded at IV, along with the wrist extensor muscle strength. The sensation in the patient’s fingers was entirely normal. Encouragingly, no recurrence of the local mass was observed during the follow-up within a span of 4 months.

## Discussion

Over the past 20 years, significant advancements in medical technology and the adoption of multi-disciplinary treatment have greatly enhanced the survival rates of patients with ARMS (7, 8). How to preserve patients’ limb function after treatment has become the next problem to be solved.

Amputation, an ancient skill, has been gradually submerged by the tide of the times while limb-sparing surgery is now the cornerstone for the treatment (9). The primary principle of surgery dictates that the ARMS must be excised in an “en bloc” way. This entails ensuring that the pseudocapsule of the tumor remains intact, without any breaches, and that the surgical boundaries encompass a wide resection area. Achieving a thorough and precise resection is absolutely crucial for successful functional reconstruction. Any attempts to reconstruct without adhering to proper surgical boundaries would simply be an exercise in futility (10). The second principle requires evaluating the extent of resection margin based on the tissue type surrounding the tumor. When a robust anatomical barrier is present, there is no need for an overly extensive resection range, and it is crucial to preserve as much normal tissue as possible (11).

For upper limb biological reconstruction, skin flaps, muscular reconstruction, ligament repair, and bone reconstruction are all vital components of these techniques. However, in this article, the focus will primarily be on the different approaches used for bone reconstruction (**Table 1**). Firstly, it should be highlighted that osteotomy is only necessary when there is involvement of bone tissue, as the bone cortex and periosteum provide an effective barrier. Blindly performing osteotomy can result in a poor prognosis (12).

**Table 1:** Surgical methods for bone reconstruction.

Surgical methods	Advantages	Disadvantages	Comments
Autogenous bone grafting	- Histocompatible and osteogenic materials - No risk of disease transmission	- Limited graft material - Donor site morbidity	- Traditional “gold standard” in bone reconstruction
Capanna technique	- Immediate rigid fixation of the graft complex - A well-perfused and osteogenic structure	- Allograft complications including graft rejection reactions	- Areliable method specifically suited for lower limb bone reconstruction
Devitalized tumor-bearing autograft	- Histocompatible - The graft is matched to the bone defect	- Unbearable risk of regional tumor recurrence - Unreliable mechanical bone strength in severely osteolytic graft	- An alternative to tumor prosthesis and allograft
Masquelet technique	- Easy to perform, no sophisticated equipment or microsurgical skills is needed	- A two-stage procedure	- Suitable in multi-tissue and infected defect
Ilizarov bone transport	- External fixation requires less soft tissue and skin - Independence from graft material	- A lengthy treatment duration and challenging nursing requirements	- Suitable in critical-sized bone defects with bad soft tissue condition
Biological 3D printing	- 100% Individualized precise customization - Unlimited supply of reconstruction materials	- The cost is high and the technology has not yet reached full maturity.	- A key future direction

Autogenous bone grafting serves a crucial purpose in bone defect repair. Among the available options, the fibula presents a preferred choice due to its compatible diameter and shape with the radius and ulna. Particularly, when the bone defect surpasses 5cm, employing vascularized fibula yields superior benefits for promoting bone healing (13). The principle of Capanna technique is similar. It increases the support of peripheral cortical bone and enhances the load-bearing and compressive capacity of bone graft composite. As a result, it is primarily utilized in the repair of lower limb bone defects (14).

Tumor-bearing segment devitalization and autograft replantation have a longstanding history in the treatment of bone defects, encompassing methods such as alcohol devitalization, microwave ablation, radiofrequency ablation, and others. However, the adoption of this method has been hindered by concerns about graft reliability. In the last decade, substantial progress in liquid nitrogen devitalization technique has emerged, holding the potential to significantly expand the utilization of this technique in bone defect reconstruction (15).

The treatment of large bone defects presents a challenge due to the contrasting growth rates between bone and surrounding tissues. This disparity often results in the bone defect area becoming filled with connective tissue, making it arduous for the bone defect to heal. However, Masquelet technique introduces a solution by employing an induced membrane that creates an optimal space for bone growth. In addition, this method generates a bioactive-rich environment, expediting the swift repair of bone defects (16).

The Ilizarov bone transport technique utilizes the principle of distraction osteogenesis, aided by an external fixator, to transfer the displaced bone segment to the site of the bone defect in a predetermined direction. This promotes the regeneration of bone tissue within the distraction gap. This technique offers significant advantages such as independence from graft material and avoidance of immune rejection or the need for risky tumor-bearing segment replantation. Additionally, it ensures optimal strength upon the completion of bone maturation. However, it is important to acknowledge the drawbacks of this method, which include a lengthy treatment duration, challenging nursing requirements, and potential complications (17).

Compared to traditional techniques, biological 3D printing technology offers several advantages. It allows for 100% individualized precise customization, enabling high-precision printing of complex anatomical structures. Additionally, it provides an unlimited supply of reconstruction materials. Consequently, although not yet widely employed in clinics, 3D printed biomaterials have garnered extensive attention and are considered the next generation of ideal biological reconstruction technology (18).

In conclusion, we present a unique case of paraulnar ARMS in the forearm, where the tumor has penetrated through the cortex of the bone and impacted the intramedullary region. Our case demonstrates that the management of ARMS necessitates the involvement of a multidisciplinary team. Neoadjuvant chemotherapy is beneficial for facilitating surgical intervention, which remains the cornerstone of treating nonmetastatic ARMS. Ensuring adequate surgical margins is paramount for effective local treatment, while implementing suitable reconstruction techniques can lead to optimal limb function. The incorporation of cutting-edge technologies like 3D printing exhibits considerable potential for transforming this field in the imminent future.

## Data availability statement

The original contributions presented in the study are included in the article/**Supplementary Material**. Further inquiries can be directed to the corresponding author.

## Ethics statement

Written informed consent was obtained from the individual(s) for the publication of any potentially identifiable images or data included in this article. Written informed consent was obtained from the participant/patient(s) for the publication of this case report.

## Author contributions

CY: Conceptualization, Resources, Writing – original draft. XW: Supervision, Writing – review & editing. HL: Resources, Writing – review & editing. JZ: Resources, Writing – review & editing. ZX: Resources, Writing – review & editing. WW: Conceptualization, Supervision, Writing – review & editing.
