# Supplementary figures and images for: Case report: A left forearm mass with eccentric intramedullary ulnar destruction diagnosed as alveolar rhabdomyosarcoma and treated by wide resection and free vascularized fibular graft
Source: Front Oncol. 2024 May 10;14:1395233. doi: 10.3389/fonc.2024.1395233 (PMC11116586; doi:10.3389/fonc.2024.1395233)

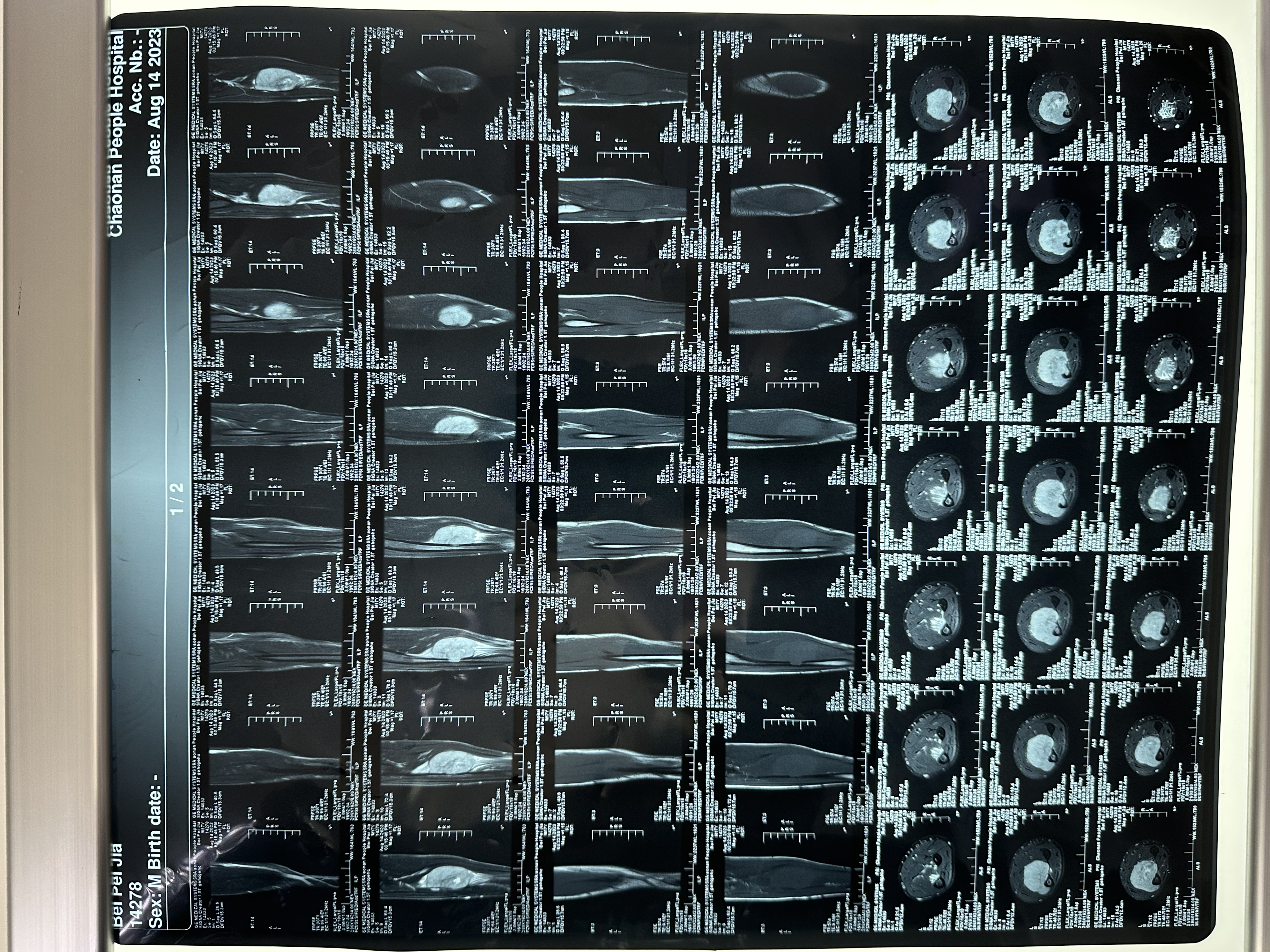

Supplement: Supplementary file 1 [file Image_1.jpeg]

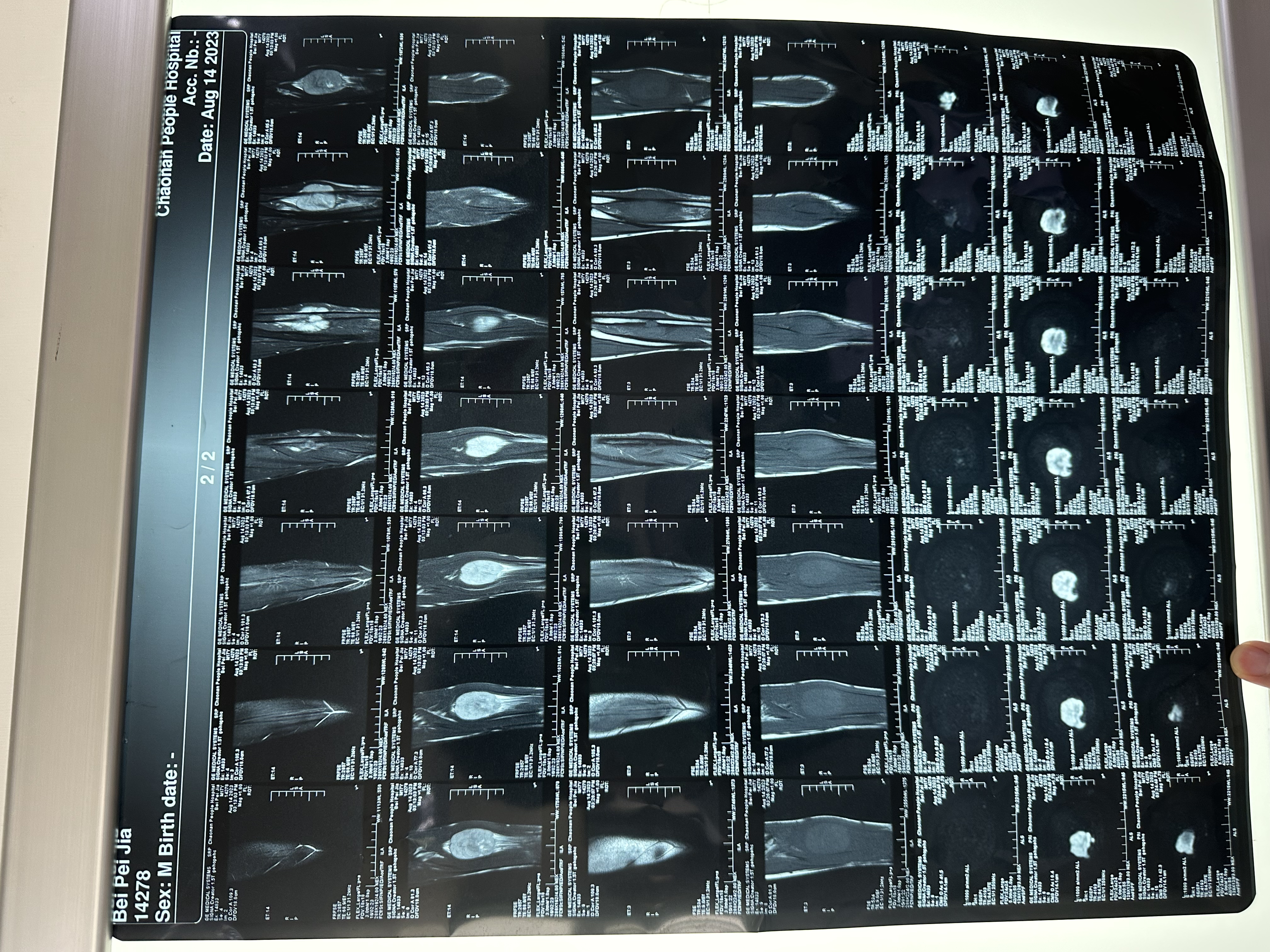

Supplement: Supplementary file 2 [file Image_2.jpeg]
